# Effect of Skeletal Muscle and Fat Mass on Muscle Strength in the Elderly

**DOI:** 10.3390/healthcare6030072

**Published:** 2018-06-26

**Authors:** Koji Nonaka, Shin Murata, Kayoko Shiraiwa, Teppei Abiko, Hideki Nakano, Hiroaki Iwase, Koichi Naito, Jun Horie

**Affiliations:** 1 Faculty of Physical Therapy, Department of Health Sciences, Kyoto Tachibana University, Kyoto 607-8175, Japan; murata-s@tachibana-u.ac.jp (S.M.); shiraiwa@tachibana-u.ac.jp (K.S.); abiko@tachibana-u.ac.jp (T.A.); nakano-h@tachibana-u.ac.jp (H.N.); horie-j@tachibana-u.ac.jp (J.H.); 2 Faculty of Physical Therapy, Department of Rehabilitation, Kobe International University, Kobe 658-0032, Japan; iwase@kobe-kiu.ac.jp; 3 Department of Physical Therapy, Hakuho College, Oji 636-0011, Japan; k.naitou@hakuho.ac.jp

**Keywords:** muscle strength, skeletal muscle mass, fat mass, grip strength, knee extension strength

## Abstract

It is important for elderly people to maintain or improve muscle strength and for clinicians to know the factors that affect muscle strength. Therefore, the purpose of this study was to compare the effects of fat mass (FM) and skeletal muscle mass (SMM) on muscle strength. The participants included 192 community-dwelling elderly women. The SMM and FM, grip strength, and knee extension strength were measured. Data were evaluated using stepwise multiple linear regression analysis, which was performed with grip or knee extension strength as a dependent variable and the SMM and FM of the upper and lower limbs as the independent variables. The SMM and FM of the upper limbs were associated with grip strength, whereas the SMM but not the FM of the lower limbs was associated with knee extension strength. These findings suggest that there may be thresholds for the SMM/FM ratio to affect muscle strength.

## 1. Introduction

Muscle strength decreases with aging [1], and this decrease is characterized by a slow walking speed [2], which in turn is associated with mortality [3]. The risk of falling and subsequently developing a fracture is reportedly associated with a decline in walking speed [4]. Therefore, it is important for elderly people to maintain or improve muscle strength.

It is well known that skeletal muscle mass (SMM) is associated with muscle strength [5]. Additionally, fat mass (FM), which is higher in the elderly than in younger individuals [6], has been shown to affect muscle strength negatively. Intramuscular adipose tissue inhibits central activation, resulting in a reduction in muscle strength [7]. It also produces tumor necrosis factor (TNF-α) [8], which reduces muscle performance [9]. The accumulation of intramuscular adipose tissue has been shown to increase with an increase in FM, as seen in individuals with obesity [10].

We hypothesized that not only SMM but also FM affects muscle strength among the elderly. If FM negatively affects muscle strength, clinicians can target it for maintenance or improvement of muscle strength in the elderly. Therefore, the aim of this study was to test this hypothesis among community-dwelling elderly women.

## 2. Materials and Methods

### 2.1. Participants

**Figure 1:**
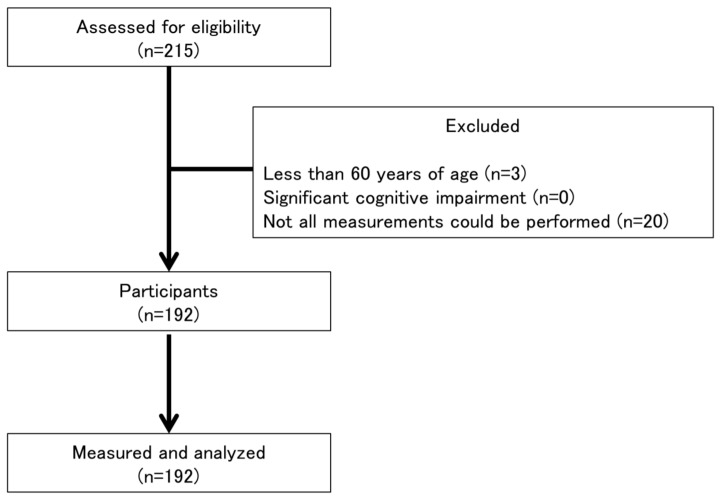
Flowchart of participation in the present study.

This was a cross-sectional study that recruited 215 participants and included 192 community-dwelling elderly women according to the following inclusion criteria (Figure 1): (1) an age of more than 60 years, (2) no significant cognitive impairment, and (3) all measurements could be performed. This study was approved by the institutional ethics committee and was conducted in accordance with the Helsinki Declaration. All participants provided written informed consent to participate in the study.

### 2.2. Body Mass Index (BMI)

The participants were grouped into different body mass index (BMI) categories in accordance with the Japanese criteria for underweight (BMI < 18.5), normal weight (18.5 ≤ BMI ≤ 24.9), and obesity (BMI > 25).

### 2.3. Exercise Habits

In order to understand their exercise habits and physical activity, participants were asked whether they performed exercise regularly for more than 30 min/day and 3 days/week.

### 2.4. SMM and FM

SMM and FM were measured by the bioelectrical impedance method using Inbody 430 (Biospace Co., Ltd., Seoul, Korea) or 470 (InBody Japan Inc., Tokyo, Japan), which were used for 107 (56%) and 85 (44%) of the participants, respectively. The participants stood on two metallic electrodes and held metallic grip electrodes, and the SMM and FM of the upper and lower limbs were measured. 

### 2.5. Grip Strength

The grip strength was measured with a hand grip dynamometer (T.K.K.58401, Takei Scientific Instruments Co., Ltd., Niigata, Japan) to assess upper limb muscle strength as described by Abe et al. [11]. The measurements were taken for both upper limbs, and the mean value was used for analysis.

### 2.6. Knee Extension Strength

The knee extension strength was measured to assess the muscle strength of the lower limbs as described by Bohannon [12]. The participants were instructed to sit with their knees and hips flexed at 90°. A hand-held dynamometer (μTas F-1; Anima Corp., Tokyo, Japan) was placed just proximal to the ankle on the anterior surface of the leg, and participants were instructed to perform maximal isometric muscle contraction. Knee extension strength was measured in both legs, and the mean value was used for analysis. 

### 2.7. Statistical Analysis

Stepwise multiple linear regression analysis was performed with grip or knee extension strength as a dependent variable and the SMM and FM of the upper and lower limbs as the independent variables. Statistical analyses were performed using SPSS ver. 22.0 (IBM Japan, Ltd., Tokyo, Japan) and significance was accepted when *p* < 0.05. 

## 3. Results

The characteristics of the participants are summarized in Table 1. The mean age, height, and weight were 73.7 ± 5.8 years, 151.5 ± 5.1 cm, and 51.0 ± 8.0 kg, respectively. In total, 66.1% of all participants regularly exercised. The percentages of underweight, normal weight, and obese were 10, 70, and 20%, respectively. The mean SMM/FM ratios of the upper and lower limbs were 1.76 ± 1.36 and 2.31 ± 1.04, respectively.

The results of the stepwise multiple regression analysis are shown in Table 2. The analysis showed that the SMM (standardized β = 0.691, *p* < 0.001) and FM (standardized β = −0.263, *p* < 0.001) of the upper limbs were independently associated with grip strength (R^2^ = 0.358). On the other hand, knee extension strength showed an independent association only with the SMM (standardized β = 0.481, *p* < 0.001) but not with the FM of the lower limbs (R^2^ = 0.231).

## 4. Discussion

The aim of this study was to test the hypothesis that muscle strength is affected not only by SMM but also by FM. In line with our hypothesis, grip strength was found to be affected by both SMM and FM of the upper limbs. On the other hand, knee extension strength was affected only by SMM but not FM of the lower limbs. These findings suggest that there may be thresholds for the SMM/FM ratio to affect muscle strength. 

The FM of the upper limb affected the grip strength. Intramuscular adipose tissue is increased with obesity and is decreased upon weight loss [10]. Our findings suggest that intramuscular adipose tissue increases as FM increases. It has been reported to inhibit central activation [7] and produce inflammatory cytokines such as TNF-α to depress myofibrillar force via the TNF receptor [9]. TNF-α has been reported to cause a decrease in the tetanic force by reducing the reactivity of muscle myofilaments to calcium [13]. Therefore, FM of the upper limbs can negatively affect grip strength by inhibiting central activation and thereby increasing TNF-α production by adipose tissue.

On the contrary, knee extension strength was not affected by the FM of the lower limbs. This could be due to less FM in lower limbs, as suggested by the decreased SMM/FM ratio in the lower limbs compared to the upper limbs. This, therefore, suggests that the FM of the lower limbs might not be large enough to affect the muscle strength. Aerobic exercise has been reported to lower FM in the lower limbs of lean (BMI < 25.0 kg/m^2^) and obese (BMI > 27.0 kg/m^2^) elderly men who did not otherwise exercise regularly [14]. Resistance training has been reported to decrease the thigh intramuscular adipose tissue in older individuals [15]. These reports suggest that regular exercise can attenuate the increase in fat tissue with aging. In this study, 66.1% of the participants exercised regularly for more than 3 days/week and 30 min/day. Therefore, intramuscular adipose tissue was expected to be low in participants in this study. Yoshida et al. reported that older adults with low intramuscular adipose tissue have normal central activation [7]. Therefore, participants in this study might not have had enough lower limb FM to affect knee extension strength, because many of them performed regular exercise. In addition, there were few cases of obesity in this study, so we could not divide the participants into underweight, normal weight, and obese groups when the effect of FM on muscle strength was assessed. Individuals with obesity may have higher intramuscular adipose tissue infiltration, and their muscle strength would thus be affected by FM.

There are two limitations of this study. First, as previously mentioned, there were few participants with obesity, so we could not determine how obesity impacts the effect of FM on muscle strength. Second, we failed to investigate the effect of FM on upper limb and lower limb activity. Further studies are therefore needed to investigate the effect of FM on muscle strength in participants divided into underweight, normal weight, and obese categories; and on the activity of the upper and lower limbs.

## 5. Conclusions

We compared the effects of SMM and FM on muscle strength. Both the SMM and FM of the upper limbs affected grip strength. However, in the lower limbs, only SMM, but not FM, was associated with knee extension strength. These findings suggest that FM can affect muscle strength when it increases to a certain degree. Therefore, clinicians may target FM to maintain or improve muscle strength in the elderly. Future studies are needed to determine the amount of FM that can affect muscle strength.

## Figures and Tables

**Table 1:** Participant characteristics.

Variable	Mean ± SD
Age (years)	73.7 ± 5.8
Height (cm)	151.5 ± 5.1
Weight (kg)	51.0 ± 8.0
Underweight (BMI < 18.5), n (%)	19 (10%)
Normal weight (18.5 ≤ BMI < 25.0), n (%)	134 (70%)
Obese (25.0 ≤ BMI), n (%)	39 (20%)
Regular exercise, n (%)	127 (66.1%)
Upper limb SMM (kg)	3.26 ± 0.65
Upper limb FM (kg)	2.17 ± 0.85
Upper limb SMM/FM ratio	1.76 ± 1.36
Lower limb SMM (kg)	10.62 ± 1.54
Lower limb FM (kg)	5.07 ± 1.54
Lower limb SMM/FM ratio	2.31 ± 1.04
Grip strength (kg)	22.1 ± 3.7
Knee extension strength (kgf)	19.9 ± 5.0
Disease	
Hypertension, n (%)	80 (42%)
Orthopedic disease, n (%)	40 (21%)
Hyperglycemia, n (%)	30 (16%)
Cardiovascular disease, n (%)	10 (5%)
Diabetes mellitus, n (%)	6 (3%)
Pulmonary disease, n (%)	5 (3%)
Rheumatoid arthritis, n (%)	3 (2%)
Renal disease, n (%)	3 (2%)
Stroke, n (%)	2 (1%)
Cancer, n (%)	2 (1%)
Others, n (%)	34 (18%)

SMM, skeletal muscle mass; BMI, body mass index; FM, fat mass.

**Table 2:** Stepwise multiple regression analysis.

Variable	β	SE	Standardized Beta	*p*-Value	R^2^	Adjusted R^2^
Grip strength					0.358	0.351
Upper limb SMM	3.889	0.384	0.691	<0.001		
Upper limb FM	−1.137	0.294	−0.269	<0.001		
Knee extension strength					0.231	0.227
Lower limb SMM	1.563	0.207	0.481	<0.001		

SMM, skeletal muscle mass; FM, fat mass.
